# Identification of long non-coding RNAs as novel biomarker and potential therapeutic target for atrial fibrillation in old adults

**DOI:** 10.18632/oncotarget.7514

**Published:** 2016-02-18

**Authors:** Yingjia Xu, Ritai Huang, Jianing Gu, Weifeng Jiang

**Affiliations:** ^1^ Department of Cardiology, Shanghai Chest Hospital, Shanghai Jiao Tong University, Shanghai, China; ^2^ Department of Cardiothoracic Surgery, Ren Ji Hospital, School of Medicine, Shanghai JiaoTong University, Shanghai, China; ^3^ Department of Cardiology, Rui Jin Hospital, School of Medicine, Shanghai Jiao Tong University, Shanghai, China

**Keywords:** long non-coding RNAs (lncRNAs), atrial fibrillation (AF), transcription factors, Gerotarget

## Abstract

Atrial fibrillation (AF) is a highly prevalent cardiac arrhythmia disease, which widely leads to exacerbate heart failure and ischemic stroke in elder world. Recently, long non-coding RNAs (lncRNAs), a subclass of noncoding RNAs, have been reported to play critical roles in pathophysiology of cardiac heart. However, little is known of their role in cardiac arrhythmia. In the present study, we investigated the expression levels of lncRNAs of AF patients and healthy people with Agilent Human lncRNA array for the first time. 177 lncRNAs of 78243 and 153 mRNAs of 30215 tested were identified to be differentially expressed (≥ 2-fold change), indicating that the expression of many lncRNAs are upregulated or downregulated in AF. Among these, NONHSAT040387 and NONHSAT098586 were the most upregulated and downregulated lncRNAs. Real time quantitative PCR were employed to validate the microarray analysis findings, and the results confirmed the consistence. GO and KEGG pathway analysis were applied to explore the potential lncRNAs functions, some pathways including oxygen transporter activity and protein heterodimerization activity were speculated to be involved in AF pathogenesis. These results shed some light on lncRNAs' physiologic functions and provide useful information for exploring potential therapeutic treatments for heart rhythm disease.

## INTRODUCTION

Atrial fibrillation (AF) is the most common heart rhythm disease in the world, accounting approximately 0.5% of the total world population [1, 2]. AF exacerbates heart failure and ischemic stroke, substantially increases the morbidity and mortality, resulting in a higher burden for patients and even the nations. However, a higher incidence of adverse consequences for the elderly has been associated with atrial fibrillation. Although AF is a heterogeneous disease, previous reports suggest that the arrhythmia may arise due to the interaction by genetic and acquired risk factors - the so-called “double hit” hypothesis [3]. Unfortunately, the precise mechanisms of atrial remodeling were not well elucidated, leading to the demand of investigating the exact mechanisms of the disease and developing treatments. Identification of novel biomarker influencing the development of AF is critical to the understanding and future prevention of the disease. Long non-coding RNAs (lncRNAs) are a subclass of noncoding RNAs (ncRNAs) and are transcribed from the genome with at least 200 nucleotides [4, 5]. The ncRNAs include microRNAs (miRNAs), PIWI interacting RNAs, and endogenous small interfering RNAs. In fact, the notion of ncRNAs acting as heart disease modulators is not new; reviews regarding the role of ncRNAs in heart disease have already been published before [6]. It has become increasingly apparent that many of the lncRNAs play molecular functions, such as controlling cell cycle, differentiation, apoptosis or as smaller RNA precursors [6-8]. LncRNAs may function through a variety of mechanisms such as modulating gene transcription by rearranging chromosomal looping and transcription factor binding. LncRNAs also affect miRNA functions by controlling pre-mRNA splicing or as miRNA sponges. Recently, accumulating evidences indicate that there is aberrant expression of lncRNAs in many heart diseases, including heart failure, pathological hypertrophy and Ventricular Septal Defect etc [9-11]. However, to the best of our knowledge, no attempts have been made to investigate the possible involvement of lncRNAs expression in AF, and the underlying pathways remain poorly understood.

## RESULTS

### LncRNAs and mRNAs expression profiles in AF

**Figure 1:**
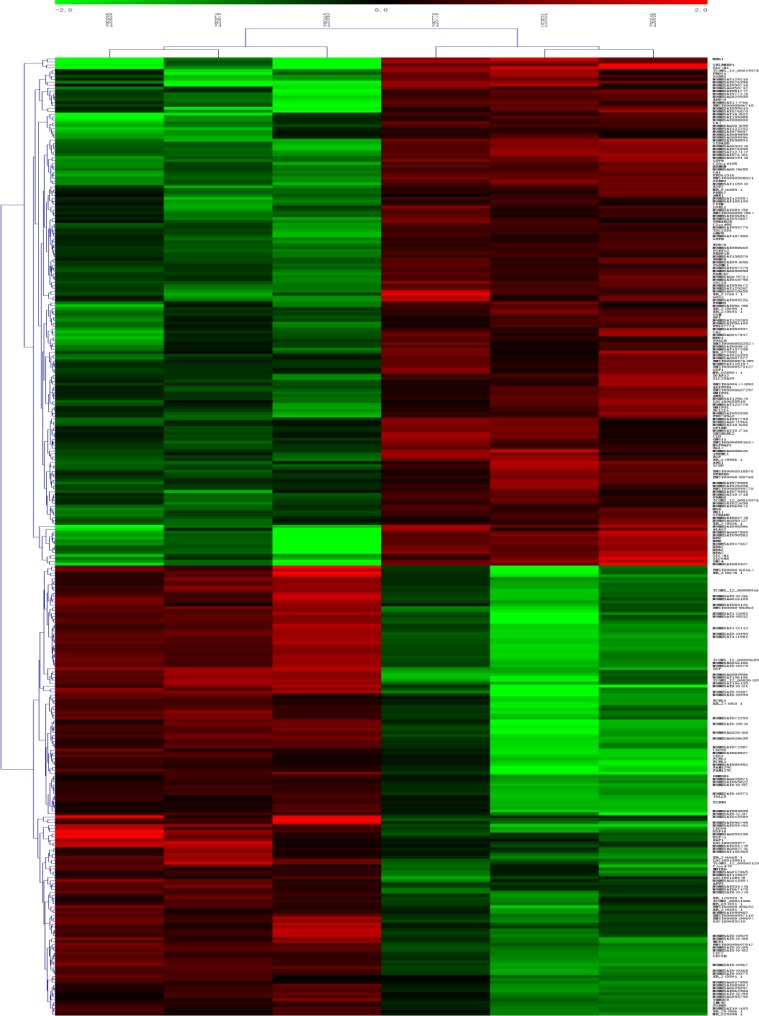
Heat map and hierarchical clustering of lncRNA profile comparison between the AF and normal blood samples Each row represents one lncRNA, and each column represents one sample. The relative lncRNA expression is depicted according to the color scale. Red indicates up-regulation; green indicates downregulation. 2.0 and −2.0 are fold changes in the corresponding spectrum, whereas left represents normal samples and right represents AF samples. The differentially expressed lncRNAs are clearly self-segregated into clusters.

LncRNA profiling showed 177 lncRNAs of 78243 tested with significant differential expression levels at least a two-fold change in AF patients compared with normal patients, with 100 up-regulated and 77 down-regulated, respectively. The top 25 differentially expressed lncRNAs were listed in Table 1. Among the dysregulated lncRNA transcripts, NONHSAT098586 is the most up-regulated, with a fold of change (FC) of 7.51, whereas NONHSAT040387 is the most down-regulated, FC being 6.94. Using the same criteria as the lncRNAs, we found that 75 up-regulated and 78 down-regulated mRNA transcripts. The most up-regulated and downregulated mRNA transcripts are hemoglobin gamma A (NM_000559) and desmoplakin (NM_004415), with FCs of 16.03 and 14.84, respectively (shown in Table 2). Hierarchical clustering of the lncRNA and mRNA profiles was performed using cluster 3.0.2; Hierarchical clustering of the expression of the 177 lncRNAs based on centered Pearson correlation clearly separated AF from normal control (Figure 1).

**Table 1:** Top 25 aberrantly expressed lncRNAs in microarray for three pairs of AF and normal blood

TargetID	FC (abs)	p	Regulation	N1	N2	N3	AF1	AF2	AF3	Chr	start	end
NONHSAT098586	7.51	0.01	up	2.70	4.54	3.94	6.32	6.76	6.83	chr4	144918491	144940455
NONHSAG007503	7.34	0.02	up	12.37	12.85	10.51	14.53	14.51	15.32	chr11	5246697	5248302
NONHSAT040387	6.94	0.01	down	6.73	6.78	7.11	2.87	4.72	4.66	chr14	106068035	107099427
NONHSAT076398	6.80	0.00	up	3.80	2.82	2.83	6.03	5.92	5.78	chr2	202316456	202323546
NONHSAT053927	6.77	0.01	up	3.29	4.28	3.13	5.91	6.07	7.01	chr17	42338172	42345509
NONHSAT098582	6.55	0.03	up	4.55	5.13	2.63	6.81	6.40	7.23	chr4	144917349	144940477
NONHSAT040421	6.52	0.03	down	5.58	6.21	6.63	2.78	4.88	2.66	chr14	106329999	107034966
NONHSAT017667	6.00	0.02	up	15.01	15.47	13.18	17.10	16.87	17.43	chr11	5246884	5248053
TCONS_l2_00030433	5.25	0.00	down	7.66	8.28	8.29	5.06	5.70	6.29	chrX	3820106	3855883
NONHSAT039492	5.21	0.01	down	10.72	10.45	9.85	7.62	8.70	7.57	chr14	96177059	96178299
NONHSAT090718	5.15	0.04	up	5.35	3.08	3.59	6.91	6.67	5.53	chr3	93624883	93630001
NONHSAG053956	5.03	0.00	down	7.97	8.40	8.26	5.63	5.64	6.36	chrX	3735557	3785832
NONHSAT098591	4.92	0.00	up	4.05	3.48	3.56	5.95	5.77	6.26	chr4	145035828	145061788
NONHSAT136136	4.81	0.00	down	7.29	7.70	7.72	4.75	5.16	6.01	chrX	3735579	3785832
XR_245045.1	4.57	0.05	down	6.44	6.89	8.40	4.12	5.71	5.34			
NONHSAT141981	4.45	0.00	down	7.40	7.30	7.92	4.88	5.95	5.33	chr16	31973408	31985682
NONHSAT142052	4.42	0.02	down	5.80	5.91	6.57	3.03	4.67	4.16	chr16	32926394	32926857
NONHSAT076870	4.35	0.05	up	6.19	5.47	3.85	7.11	6.90	7.87	chr2	218678431	218680091
NONHSAT040390	4.32	0.00	down	10.72	10.84	11.13	8.30	9.24	8.81	chr14	106111027	106478375
ENST00000460164	4.18	0.03	down	13.24	12.64	13.65	9.98	11.83	11.51	14	106110833	106114892
ENST00000456563	4.10	0.00	down	8.76	9.10	9.22	6.43	7.09	7.46	X	3772390	3785176
NONHSAT040540	4.02	0.02	down	6.48	7.04	7.05	4.03	5.79	4.73	chr14	107108251	107108696
NONHSAT076401	3.98	0.00	up	5.85	5.87	5.55	7.86	7.45	7.95	chr2	202343864	202345071
NONHSAT136135	3.98	0.00	down	7.64	8.26	8.03	5.62	6.10	6.23	chrX	3735575	3742639
NONHSAT055019	3.95	0.03	up	4.35	2.68	2.68	5.42	4.76	5.47	chr17	57695918	57696926

**Table 2:** Top 25 aberrantly expressed mRNAs in microarray for three pairs of AF and normal blood

Genbank Accession	Gene Name	FC (abs)	p	Regulation	N1	N2	N3	AF1	AF2	AF3
NM_000559	HBG1	16.03	0.01	up	4.97	7.33	4.25	9.98	9.40	9.18
NM_000342	SLC4A1	14.84	0.00	up	3.15	4.55	2.77	7.31	6.75	8.09
NM_003944	SELENBP1	9.25	0.02	up	4.31	5.50	2.77	6.99	6.99	8.22
DQ656067	clone Affy2H6-6	8.66	0.02	up	4.87	6.87	4.49	8.68	8.77	8.11
NM_000519	HBD	6.24	0.02	up	14.37	15.04	12.74	16.55	16.53	16.99
NM_000517	HBA2	6.15	0.02	up	13.82	13.80	11.72	15.40	15.75	16.05
NM_004415	DSP	6.09	0.00	down	5.35	5.51	5.75	2.93	3.09	2.77
XM_003119591	uncharacterized LOC100508797	5.80	0.04	down	7.52	6.98	7.42	3.18	5.75	5.39
NM_007308	SNCA	5.76	0.01	up	10.48	10.84	9.37	12.80	12.44	13.04
NM_000518	HBB	5.75	0.03	up	15.36	15.64	13.28	17.22	17.09	17.54
NM_000032	ALAS2	4.88	0.04	up	6.45	8.03	5.64	8.44	9.55	8.99
AY172958	anti-rabies SOJB immunoglobulin heavy chain	4.87	0.03	down	10.42	10.10	11.64	7.39	9.17	8.75
NM_017414	USP18	4.60	0.01	down	7.63	6.71	6.10	4.15	5.01	4.67
HCG1981372		4.29	0.00	down	8.17	8.80	8.76	6.25	6.25	6.95
XM_003403505	Ig kappa chain V-III region VH-like	4.27	0.01	down	9.61	9.56	9.87	6.65	8.18	7.93
AY505570	Immunoglobulin heavy chain	4.22	0.00	down	9.56	9.36	10.13	7.16	8.09	7.57
A_33_P3331178		4.21	0.02	down	7.63	7.74	8.20	5.28	6.77	5.29
A_24_P110242		3.99	0.03	down	9.48	9.72	10.90	7.39	8.80	7.93
NM_002100	GYPB	3.93	0.00	up	3.44	3.56	3.67	5.33	5.53	5.73
XM_003403854	Ig heavy chain V-III region VH26-like	3.92	0.01	down	10.49	10.46	10.82	7.74	9.06	9.06
XM_003403466	Ig heavy chain V-I region V35-like	3.86	0.01	down	11.01	10.85	11.47	8.56	9.63	9.30
Z46771	Immunoglobulin heavy chain VDJ region	3.85	0.00	down	9.15	9.07	9.73	6.94	7.87	7.31
L03830	Ig rearranged H-chain mRNA V-region	3.84	0.01	down	8.62	8.70	8.86	6.21	7.50	6.66
Z18824	Rearranged Ig H-chain V-domain	3.83	0.01	down	8.56	8.75	9.56	6.46	7.65	6.95
AY393117	Clone RA702-M1-22.fa Immunoglobulin heavy chain	3.78	0.01	down	9.71	9.68	10.13	7.40	8.51	7.85

### Validate the results of microarray by qPCR

**Figure 2:**
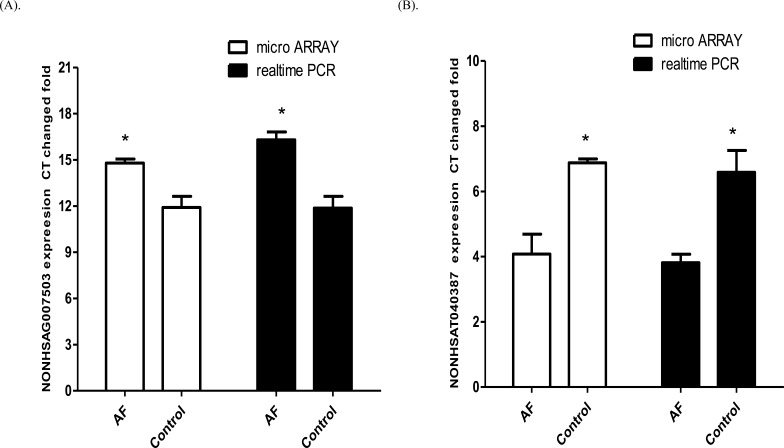
Comparisons between microarray data and qPCR results NONHSAG007503 **A.** and **B.** NONHSAT040387 were differentially expressed in AF samples compared with control samples detected by microarray and real-time quantitative PCR. The validation results of the lncRNAs indicated that the microarray data correlated well with the qPCR results. The results are presented as mean ± SEM (*n* = 3 in micro array, *n* = 10 in realtime PCR). *p < 0.05 and ** *p* < 0.01 *vs* corresponding controls.

LncRNAs transcripts were validated by quantitative PCR with 30 human blood samples (20 AF samples and 10 control samples). Two lncRNAs (NONHSAG007503 and NONHSAT040387) were randomly selected to prove the consistency of microarray and qPCR. As expected, the expression of lncRNA NONHSAG007503 was up-regulated and NONHSAT040387 was down-regulated in the AF samples versus control samples (Figure 2), consistent with the microarray results.

### Go and pathway analysis

**Figure 3:**
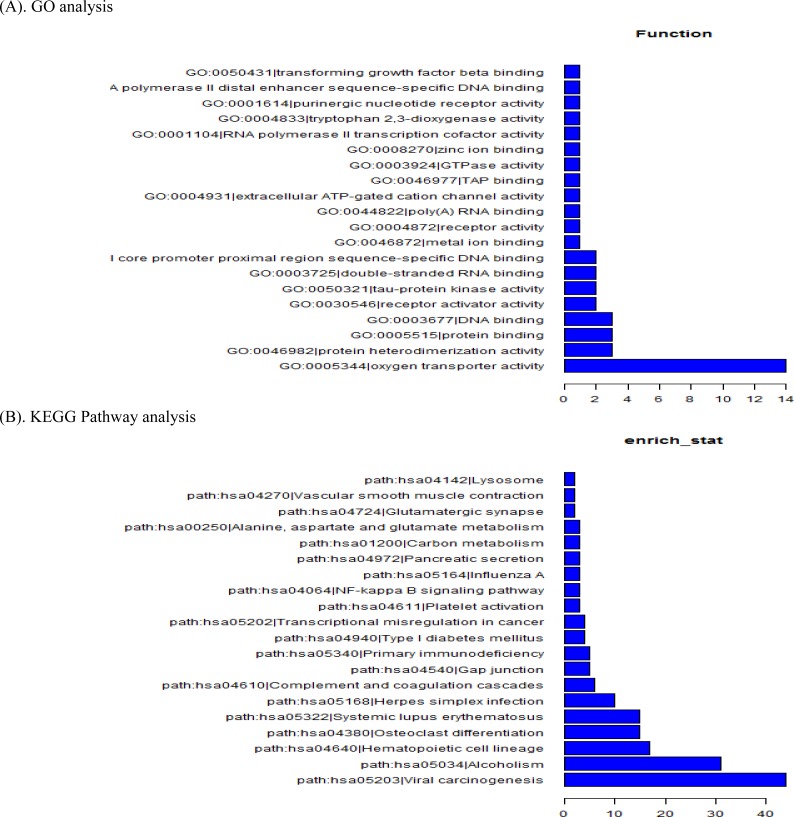
GO analysis **A.** and KEGG Pathway analysis **B.** of aberrantly expressed lncRNAs in AF.

To predict the functions of the lncRNAs, we adopted methods originally demonstrated in previous reported paper [12]. Generally, The GO category was classified by Fisher's exact test, and the *p*-value was corrected by the false discovery rate (FDR) calculation. The presenting key genes in gene networks and canonical pathways were identified by the curated Ingenuity Pathway Analysis (IPA) database according to KEGG. The enriched functional terms were used as the predicted functional terms for each given lncRNAs. GO analysis indicated that several functional pathways were enriched. Among these pathways, oxygen transporter activity, protein heterodimerization activity, and DNA binding were the most closely associated with AF (Figure 3A). Furthermore, using the same criteria as the GO analysis, KEGG Pathway analysis showed some corresponding pathways, including viral carcinogenesis, alcoholism, hematopoietic cell lineage, osteoclast differentiation and complement and coagulation cascades, etc. (Figure 3B).

### Construction of co-expression network

**Figure 4:**
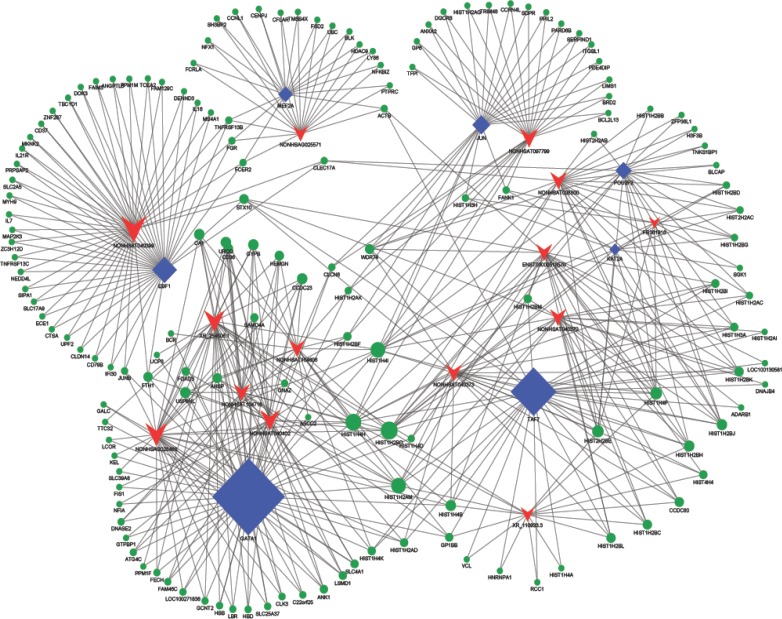
LncRNA-mRNA-network was constructed based on the correlation analysis between the differential expressed lncRNAs and mRNAs In the network, blue node represents Transcription factors, red node represents the lncRNAs, and green node represents the target mRNAs. The size of node is proportional to the outgoing link number. The thickness of outgoing link represents statistical relationship to the number of occurrences of the results proportionally.

To explore which lncRNAs and mRNAs play critical roles in AF progression, we constructed a co-expression network of the differentially expressed correlated lncRNAs and mRNAs. The correlation between lncRNAs and mRNAs was expressed with Pearson's correlation coefficients and those no less than 0.99 were used to construct the network. Transcriptional regulatory elements may exist in non-coding regions, but it could be tough to distinguish these only guiding by primary sequences. To explore lncRNAs that possibly have trans-regulating functions, we compared the mRNAs that coexpressed with these lncRNAs with those mRNAs including regulatory targets of certain Transcription factors (TFs). It can be helpful to accurately map the boundaries of regulatory elements because of the narrow transcription factor binding sites. As shown in Figure 4, GATA1 closely correlated with many mRNAs and lncRNAs. Similar results were showed in TAF7 and EBF1, indicating the three transcriptional regulatory elements may play critical roles in lncRNAs process.

## DISCUSSION

AF is the most common cardiac diseases, bringing huge burdens to old patients and their families. The prevalence of AF increases rapidly with age. However, the exact pathogenesis and serum biomarkers of AF are still not well elucidated. Sensitive serum biomarkers reflect the development of atrial remodeling, thus it could be simpler to determine the state of atrial remodeling and perform interventional therapy timely through observing serum biomarkers [13]. However, so far the correlation among lncRNAs, atrial remodeling and serum biomarkers remains unknown. To the best of our knowledge, this study is the first comprehensive lncRNAs analysis with AF in blood. This study was designed to discover the relationship between lncRNAs expression and atrial structural remodeling of AF. These data indicated that 177 lncRNAs displayed significant differential expression in AF. Functional elements are always identified by extreme conservative evolutionary sequences, but many lncRNAs are poorly conserved though they play roles in the heart. Ruan et al has discovered 219 lncRNAs differentially expressed in atrial tissues between AFs and controls [14]. Our study results are consisted with Ruan's study. For example, GO analysis found same changes in molecular function between AF compared with controls, including DNA binding, protein binding, metal ion binding and transforming growth factor beta binding. Differential expression of lncRNAs may be affected by AF and atrial remodeling and our work has identified the differentially expressed lncRNAs in AF. However, it needs further investigation to confirm the relationship between their expression and function. Subgroup analysis of lncRNAs should be performed to further exploration on the regulatory network. Unfortunately, available experimentally verified lncRNA associations are still comparatively rare, further functional studies are required to elucidate their roles in AF. These findings bring profound influences on cardiovascular science research and provide golden opportunities for intervention therapies in disease progression.

Cardiac science including development and adaptation regulatory networks has been intensively investigated. In the past ten years, with the help of development in molecular and biotechnology, scientists have achieved great progress in elucidating the molecular mechanisms of heart formation. In recent years, genetics research based on family and population showed that transcription factors may play important roles in arrhythmia susceptibility [14-16]. Moreover, animal studies have demonstrated that transcription factors performed crucial roles in atrial remodeling, indicating the importance of transcription factors in AF. In the present study, we found that several transcription factors such as GATA1, TAF7 and EBF1 could be essential for lncRNAs expression in AF development. Pathway identification showed that GATA1, TAF7 and EBF1 played central roles in AF, which were consistent with previous reports [17,18]. Close physical links between lncRNAs and developmental functional genes does not necessarily indicate that there would be functional links between protein-coding genes and lncRNAs. For example, recent researches on mice suggested that there were no evident correlations between expression levels of lncRNAs and their adjacent genes [19-22]. Therefore, further researches are demanded to fully elucidate the molecular mechanisms between these transcription factors and AF pathogenesis. In the future, such researches can help making significant progress in the clinical treatment of arrhythmia.

In conclusion, we explored and found out the dysregulated expression of lncRNAs in human AF for the first time. These data suggest that a great variety of lncRNAs are involved in AF development and present background/reference resources for future exploring the functions of lncRNAs in AF development. More investigation will be required to define the physiologic functions and the mechanisms by which these lncRNAs affecting AF formation.

## MATERIALS AND METHODS

### Ethics statement

This research was permitted by the human ethics committee of the Shanghai Chest Hospital, People's Republic of China. All AF patients and health control people have been informed with written consent to use their blood samples for this study. The AF patients were elder adults (age=55±5 years), without hypertension, diabetes, hyperthyroidism and other heart disease.

### Blood collection and RNA extraction

5 ml blood of each person was collected and immediately stored at 4°C until use. According to the manufacturer's protocols, RNA Isolation Kit (Ambion, USA) was used to extract total RNA from blood samples within 24h after collection. Total RNA was quantified with the NanoDrop ND-2000 (Thermo Scientific, USA) and the RNA integrity was assessed using Agilent Bioanalyzer 2100 (Agilent Technologies, USA).

### LncRNA and mRNA microarray expression profiling

Agilent Human LncRNA Microarray v 4.0 is designed for the analysis of global human lncRNAs and protein-coding transcripts. The microarray profiling was conducted in the laboratory of the OE Biotechnology Company in Shanghai, People's Republic of China. The sample labeling, microarray hybridization and washing were performed as described by the manufacturer. In brief, total RNA were transcribed to cDNA, synthesized into cRNA embedding with Cyanine-3-CTP. These tagged cRNAs were hybridized onto the Human lncRNA array, including a global profiling of 78,243 human lncRNAs and 30,215 coding transcripts. The arrays were scanned with the Agilent Scanner G2505C (Agilent Technologies, USA) after washing. Array images were analyzed by Feature Extraction software (version 10.7.1.1, Agilent Technologies) and the raw data were extracted. The raw data were further analyzed by Genespring (Version 12.5, Agilent Technologies). The raw data were firstly normalized, setting a change threshold>2.0 and *p* value<0.05 for up- and down-regulated genes. Then, Hierarchical Clustering was employed to calculate the distinguishable lncRNA and mRNA expression patterns.

### Functional group analysis

The functions in biological pathways or GO terms of these closest coding genes were analyzed by Pathway and GO analyses Kyoto Encyclopedia of Genes and Genomes (KEGG) analysis according to the latest KEGG database (http://www.genome.jp/kegg/) was employed to determine the biological roles of these differentially expressed mRNAs. Significance is judged when p value (Hypergeometric-P value) is less than 0.05.

### Co-expression network construction

To discover the potential targets of lncRNA, we analyzed the interaction between lncRNAs and corresponding transcription factors based on hypergeometric cumulative distribution function with the help of MATLAB 2012b (The MathWorks, USA). The graph of the lncRNAs-TFs network was drawn with the help of Cytoscape 3.01 (Agilent and IBS, USA). If the intersection of these two groups is large enough (*p* < 0.01, calculated by hypergeometric cumulative distribution function and FDR < 0.01, under the control of the Benjamini and Hochberg procedure), then we predict that these lncRNAs possibly participate in pathways regulated by these TFs. The recently released ENCODE data on TFs and their regulatory targets were used in our analysis

### Real-time quantitative reverse transcription PCR

A two-step reaction process was used for quantification reverse transcription [21] and PCR. Each RT reaction consisted of 0.5 μg RNA, 2 μL of Primer Script Buffer, 0.5 μL of oligo dT, 0.5 μL of random 6 mers, 0.5 μL of Primer Script RT Enzyme Mix I (TaKaRa, Japan) and nuclease-free water to reach a volume of 10 μL. Reactions were performed in the GeneAmp^®^ PCR System 7500 (Applied Biosystems, USA) for 15 min at 37°C, then inactivation of RT by heating at 85°C for 5 s. Then the RT mix was diluted by 10-fold with nuclease-free water and stored at −20°C. While running real-time quantitative PCR, melting curve was analyzed to verify the specificity of the aimed PCR product. All experiments were done in triplicate. Glyceraldehyde-3-phosphate dehydrogenase was used as an endogenous control to normalize and using the 2-ΔΔCt method for lncRNAs expression calculation. The primer sequences were designed in the laboratory based on the DNA sequences and is shown: NONHSAG007503 (forwards primer GGAGAAGTCTGCCGTTAC; reverse primer TCAAAGAACCTCTGGGTCC) and NONHSAT040387 (forwards primer CTTCAGTAGCTCTGCTATGC; reverse primer AGAGTCTGCGTAGTATATGGTA).

### Statistical analysis

All results were represented as the means ± SD or proportions. For comparisons, paired t-tests and unpaired t-tests were performed where appropriate. All graphs were plotting using GraphPad Prism 5.0 for Microsoft Windows (GraphPad Software, USA). Two-sided *p*-values were calculated by the SPSS 16.0 (SPSS, USA) and statistical significance was judged when *p* < 0.05.

## SUPPLEMENTARY MATERIAL FIGURE


