# Supplementary material for: Identification of long non-coding RNAs as novel biomarker and potential therapeutic target for atrial fibrillation in old adults
Source: Oncotarget. 2016 Feb 18;7(10):10803–11. doi: 10.18632/oncotarget.7514 (PMC4905440; doi:10.18632/oncotarget.7514)
Supplement: Supplementary file 1 [file oncotarget-07-10803-s001.pdf]

Identification of long non-coding RNAs as novel biomarker and potential therapeutic target for atrial fibrillation in old adults

Supplementary Material

(A). GO analysis - Component

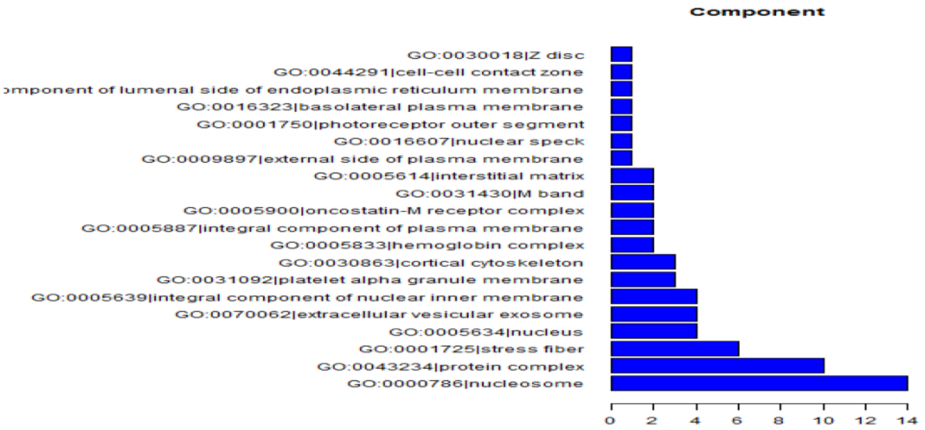

(B). GO analysis - Process

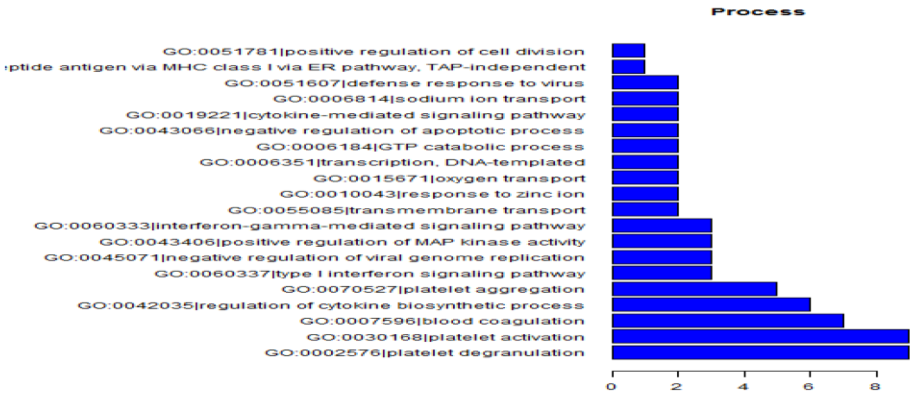

Supplementary Figure 1: GO analysis aberrantly expressed lncRNAs in AF
